# Do Transit-Oriented Developments (TODs) and Established Urban Neighborhoods Have Similar Walking Levels in Hong Kong?

**DOI:** 10.3390/ijerph15030555

**Published:** 2018-03-20

**Authors:** Yi Lu, Zhonghua Gou, Yang Xiao, Chinmoy Sarkar, John Zacharias

**Affiliations:** 1 Department of Architecture and Civil Engineering, City University of Hong Kong, Hong Kong, China; 2 City University of Hong Kong Shenzhen Research Institute, Shenzhen 518057, China; 3 School of Engineering and Built Environment, Griffith University, Gold Coast, QLD 4215, Australia; z.gou@griffith.edu.au; 4 Department of Urban Planning, Tongji University, Shanghai 200092, China; yxiao@tongji.edu.cn; 5 Healthy High Density Cities Lab, HKUrbanLab, The University of Hong Kong, Hong Kong, China; csarkar@hku.hk; 6 College of Architecture and Landscape, Peking University, Beijing 100871, China; johnzacharias@pku.edu.cn

**Keywords:** transit-oriented development (TOD), walking, new towns, urban planning, physical activity, transportation

## Abstract

A sharp drop in physical activity and skyrocketing obesity rate has accompanied rapid urbanization in China. The urban planning concept of transit-oriented development (TOD) has been widely advocated in China to promote physical activity, especially walking. Indeed, many design features thought to promote walking—e.g., mixed land use, densification, and well-connected street network—often characterize both TODs and established urban neighborhoods. Thus, it is often assumed that TODs have similar physical activity benefits as established urban neighborhoods. To verify this assumption, this study compared walking behaviors in established urban neighborhoods and transit-oriented new towns in Hong Kong. To address the limitation of self-selection bias, we conducted a study using Hong Kong citywide public housing scheme, which assigns residents to different housing estates by flat availability and family size rather than personal preference. The results show new town residents walked less for transportation purpose than urban residents. New town residents far from the transit station (800–1200 m) walked less for recreational purpose than TOD residents close to a rail transit station (<400 m) or urban residents. The observed disparity in walking behaviors challenges the common assumption that TOD and established urban neighborhoods have similar impact on walking behavior. The results suggest the necessity for more nuanced planning strategies, taking local-level factors into account to promote walking of TOD residents who live far from transit stations.

## 1. Introduction

### 1.1. Physical Activity and Health

Many Chinese cities have undergone exceptionally rapid expansion and sprawl since economic reforms began in 1978 [1]. Insufficient pedestrian infrastructure and pedestrian-oriented destinations, underfunded public transportation services, and immense urban blocks in many developments during recent decades may partially contribute to rapid decline in physical activity and marked increase of obesity rates [2,3]. From 1991 to 2006, urban residents’ physical activity level fell by 32% [3]. The obesity rate has already reached 20% in some Chinese cities, comparable to those in developed countries [4]. China has surpassed the United States with most obese population of 89.6 million adults according to a study in 2016 [5].

Physical inactivity in these cities is therefore a major public health challenge with documented risks of non-communicable diseases (e.g., type II diabetes, metabolic syndrome, and cardiac heart disease) and surging societal healthcare burden. There is strong evidence that physical activity, such as walking and cycling, can benefit adults in numerous ways, including the prevention and treatment of chronic illnesses, and improved physiological and psychological health [6]. Many local governments, therefore, are preparing policies to stimulate walking in their health and disease prevention strategies.

### 1.2. What is Transit-Oriented Development?

For the last two decades, urban planners and public health officials in western countries have recognized the importance of urban built environment on walking and leisure-related physical activity, such as outdoor running and bicycling [7,8,9,10,11,12,13]. The concepts of transit-oriented development (TOD), new urbanism, and smart growth emerged with such efforts [14,15].

TOD is a response to support walking within acceptable distances—e.g., 800 m, or about 10 min walking time—by designing high-density, mixed-use, pedestrian-friendly urban areas around public transit. Many studies have demonstrated that residents in TOD are more physically active compared with those in car-oriented urban sprawl [16,17,18,19]. Yet it is unclear whether residents in TOD are equally physically active compared with those in old established urban neighborhoods.

### 1.3. Hong Kong New Town Development

**Figure 1:**
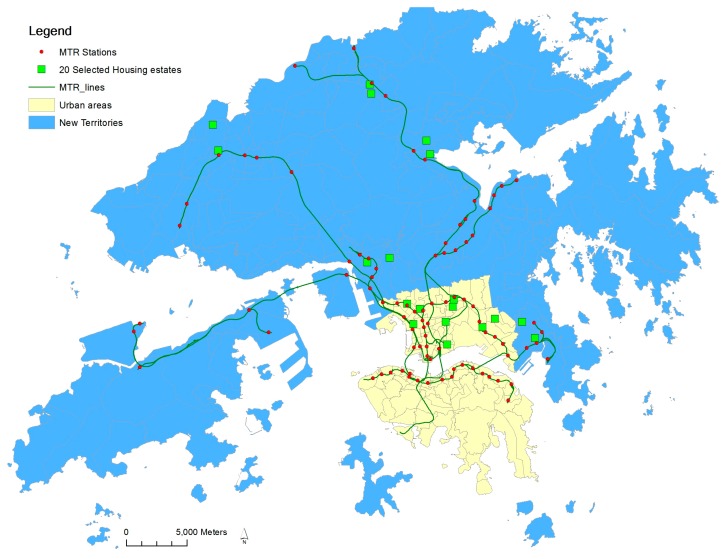
The 20 estates in Study 2 are shown, along with the urban areas established before 1970 and New Territories in Hong Kong. The New Territories feature nine transit-oriented development (TOD) new towns, which accommodate half of the population.

Hong Kong comprises three major parts: Kowloon, Hong Kong Island, and New Territories. It is inherently a hybrid city characterized by traditional compact urban neighborhoods in Kowloon and Hong Kong Island and transit-oriented new towns in the New Territories. Urban developments were built up before the WWII era and are concentrated in Hong Kong Island and Kowloon. Those developments often achieved high density, mixed land-use, and gridiron street networks. New Territories, comprising 95% of total land area of Hong Kong and originally occupied by open rural areas and farm lands, were developed in late 1950s to 1970s (Figure 1). The new towns’ development in New Territories is an adaptation of the British new town concept of the post-WWII era, in fact, predating the TOD concept developed in the US [20,21]. Those new towns, however, have all the characteristics of TOD concepts, such as high-density, mixed-use, pedestrian orientation around a transit station, through their size and density exceeds many examples from the US. Therefore, those new towns are often recognized as example of TOD in high-density cities, acting as a microcosm for replication in other Chinese cities, due to extremely dense population and limited land resources [21,22,23]. Until now, about half of the population of Hong Kong are living in new towns in New Territories while the rest live in urban areas [24].

**Figure 2:**
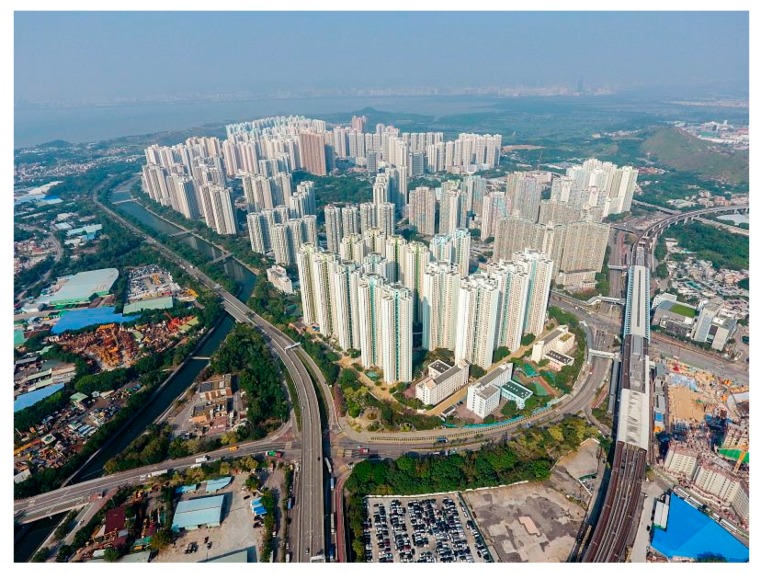
The aerial view of Tin Shui Wai in 2016 [26]. It is a typical Hong Kong new town, confined by open rural areas and connected to other districts with mass transit railway (MTR) and other public transportation services.

**Figure 3:**
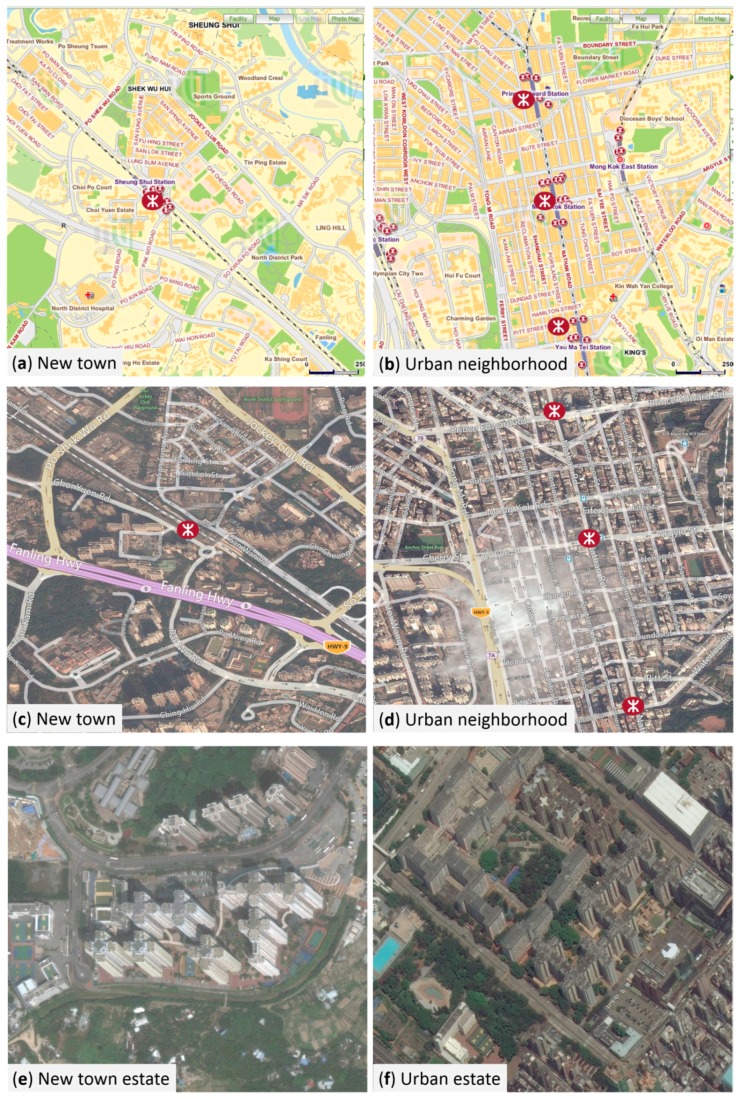
The Hong Kong new towns are often confined by surrounding open areas (**a** & **c**). The established urban areas are continuously developed, and different parts are immediately adjacent to each other (**b** & **d**). The new town housing estates far from MTR stations are adjacent to open rural areas (**e**), while urban housing estates far from MTR stations are still surrounded by dense urban developments (**f**). Legend: red dots represented MTR stations. (Source: Microsoft Bing maps.).

The New Territories in Hong Kong feature linear clustering of new towns along the mass transit railway (MTR) lines. These new towns are often separately developed and confined by surrounding rural open spaces (Figure 2 and Figure 3). A new town has almost all the physical characteristics of TOD: mixed land use, high urban density, and well-connected streets around rail transit stations, although not necessarily in gridiron layout. The city-wide transit system consists of different modes, mainly railways, road-surface trams, buses, and ferries. These systems provide its new town residents with a high level of mobility. The convenient transit system, together with high cost of owning private automobiles keeps the private automobile ownership extremely low compared with other developed societies (0.07 cars per person as of 2015) [25]. The core commercial and business developments are often densely built around MTR stations, and gradually become scant with increased distance from them.

The old established urban areas, on the other hand, are more evenly developed than contemporary new towns. The urban neighborhoods also feature mixed land-use, high density, and walking-friendly design features. More importantly, these neighborhoods connect to adjacent neighborhoods and areas and form a much larger continuous walk-supported environment. Commercial and business developments are evenly distributed throughout the whole region, rather than closely clustered around the MTR stations (Figure 3).

### 1.4. Findings of Previous Research 

Numerous studies have found that people in transit-oriented development (TOD) neighborhoods have better chance to reach the recommended daily physical activity [16,18] and less likely to be overweight and obese [27] than those in auto-oriented neighborhoods. For example, approximately 20% TOD residents met the physical activity recommendation through their utilitarian trips. While only 5% of general residents achieve the recommended levels. It suggested that living in a TOD promotes physical activity [16].

The residents in TOD neighborhoods have been known to rely more on transit use or walking than those in car-oriented neighborhoods [19,28,29]. Walking primarily associated with public transit use, such as walking to from transit stations, is proven to be a major source of daily physical activity [30,31]. Daily physical activity level was higher on days on which people used transit compared with days when they did not (47.6 vs. 56.3 min). The difference in physical activity can be entirely explained by transportation walking [30].

These findings suggest that certain built environment features associated with TOD facilitate physical activity by encouraging transportation walking and transit use. The key features can be summarized within the framework of the “3Ds”: land use diversity, density, and pedestrian-friendly design [32] or “5Ds”: adding distance to transit stops and destination accessibility [33] or “3D + R”: density, distance, destination + route [34].

### 1.5. Gaps in the Research

Supported by increasing empirical studies which often observed physical activity benefits in western countries, TOD has already attracted significant attention in new town or suburban planning in China [35]. Hong Kong is often used as a measuring stick for how the TOD concept can be replicated in Chinese cities. Yet three major gaps may limit the application of TOD in China.

(1) Earlier studies comparing travel behaviors between TOD and car-oriented neighborhoods often use established urban neighborhoods as proxies for TOD neighborhoods, because of the scarcity of TOD neighborhoods in 1990s [19,36,37]. Those researchers implicitly assumed that TOD neighborhoods and established urban neighborhoods have similar impact on walking potential and physical activity levels. Indeed, TOD and established urban neighborhoods sharing many similar walking-supporting design characteristics. However, such assumption that the travel behaviors are the same in TOD neighborhoods and established urban neighborhoods is unverified, especially in the context of Chinese cities, for the established urban neighborhoods in those cities differ from TOD neighborhoods in some respects. The first difference is *density continuity*. For example in Hong Kong, new towns are often contained by surrounding open rural areas, while the urban areas are continuously developed throughout coastal areas (Figure 2 and Figure 3). The second difference is *job density*. Urban areas in Hong Kong Island and Kowloon, serving as the central business center, have more job opportunities than new towns.

Thus, we hypothesize that the residents of new towns may have lower walk levels due to such environmental and economic differences, compared with established urban neighborhoods in Hong Kong. Furthermore, we expect the open-space-bounded new towns may exhibit an edge effect on walking behavior of residents; as the peripherally located new town residents living far from transit stations receive less exposure to TOD design features, and in turn, walk less compared with core new town residents living close to transit stations. Meanwhile, the continuous developed old urban neighborhoods have no such edge effect. The urban residents living far from transit stations may still benefit from 3D design features and pedestrian infrastructure, thereby having similar levels of walking compared with urban resident living close to transit stations.

(2) Most of these studies of the impact of TOD have been conducted in the US and other Western countries. By comparison, the local urban and social contexts in China markedly differ from those in Western countries, such as cities in China typically have higher urban population densities, more established public transportation systems, and lower household income and lower private car ownership. Some emerging studies have been investigated the travel behavior of TOD residents in some major Asian cities, and they found that TOD residents may have longer commute time than established urban residents due to the lacking of job opportunities in TOD new towns [35,38,39,40,41]. Yet the concept of TOD has already been widely accepted in China. To understand the influence of TOD design on people’s walking behavior will have immense impact on the long-term physical activity and health of a broad mass of the population.

(3) The design of most previous neighborhood-level cross-sectional studies are often limited by the possible bias of residential self-selection; that individual factors may be related to both the physical activity and residential choices [42,43,44]. For example, a person who appreciates the benefit of walking may move to a neighborhood supporting walking, and hence walk more in that neighborhood. Residential self-selection thus may alternatively explain any observed physical activity-built environment associations in cross-sectional research [45].

To reduce the residential self-selection bias, random assignment of residence is an essential but rarely implemented research component. The citywide public housing system in Hong Kong provides an exceptional opportunity for a natural experiment that largely overcomes self-selection bias. Low-and medium-income families can apply for living in public housing flats with low rent. Applicants are in most instances assigned to available public housing estates due to limited number of available public housing flats. The applicants are allocated by random computer batching according to applicant’s family size and prevailing public housing allocation standards, albeit elderly applicants are given priority in housing allocations over others [46]. Therefore, the present study provides an alternative way to residential self-selection bias.

### 1.6. The Current Study

With the objective of addressing the above-mentioned issues and provide a clearer understanding of the role of TOD on walking behavior in a Chinese context, the present study focused on walking behaviors of public-housing residents randomly assigned to traditional urban or TOD neighborhoods, and neighborhoods close to or far from MTR stations in Hong Kong. Furthermore, the attributes of built environment in the respective public housing estates were objectively analyzed with Geographic Information Systems (GIS).

## 2. Methods

Hong Kong is a high-density city located on the southeast coast of China with a land area of 1104 km^2^. Its population reaches 7.3 million in 2015. Hong Kong is known for its new town developments, characterized with TOD features, which were initiated in 1970 as a response to a large influx of immigrants from mainland China. Hong Kong is also well known for its citywide public housing schemes providing affordable accommodations for its low-income residents. In 2015, there were about 2 million residents in 160 public housing estates across all districts [24].

### 2.1. Study Area

We selected 20 public housing estates with two criteria: neighborhood type (categorized as traditional urban vs. TOD) and distance to Mass Transit Railway (MTR) station (close vs. far). Public housing estates located in urban area of Kowloon were classified in the traditional urban neighborhood group; those located in new towns developed since 1970 in the New Territories were classified into the new town group [21]. Given the extremely dense population, the dense areas of new towns in Hong Kong, as well as many TODs in China, often go beyond the suggested 800 m buffer area of MTR stations. The dense areas of new towns, such as high-rising housing estates, are commonly with 1200 m buffer area. Yet, people still heavily relay on public transportations systems. The population census data revealed that 81.1% of residents within 800–1200 m from a MTR station use public transportation system for commuting trips, while 82.7% of residents within 400 m from a MTR station do so [24]. Therefore, public housing estates within 400 m walking distance were classified in the close group; those within 800–1200 m were classified in the far group.

The 20 selected public housing estates were stratified into four groups with five estates in each group: urban/close to MTR, urban/far from MTR, TOD/close to MTR, and TOD/far from MTR (Figure 1). Given the research hypothesis, effort was made to assure public housing estates in the far group are on the outskirts of TOD new towns, i.e., they are immediately adjacent to surrounding open rural spaces confining new towns.

The medium household income of house estates was also included as an additional selection criterion, for family socioeconomic status (SES) may also shape travel behavior and account for differences in the level of physical activity. Only the housing estates with a medium monthly household income within HK$12,000–HK$15,000 were selected in the present study.

### 2.2. Study Participants

The study assessed 30–35 adults from each housing estate, with a total of 616 study participants. The participants were Chinese adults aged 18 to 64 years, who can perform physical activity independently and had lived in the estate for more than one year. The research team selected the participants by convenience in 2016. In each housing estate, we approached residents leaving or entering the housing estate. Ethical approval for the study was obtained from the Research Committee of City University of Hong Kong (H000691).

### 2.3. Covariates 

During face-to-face interviews, the research team collected participants’ SES—measured by household income (<HK$12,000, HK$12,000–HK$15,000, ≥HK$15,000)—and demographic characteristics including sex and age. The participants’ ages were transformed into a categorical variable with three levels (18–34 years, 35–49 years, and 50–64 years). Other built environment factors may be also associated with walking [9,47,48,49,50]. They include intersection density, land-use mix, and population density; they were assessed within 800 m straight-line buffers around house estates in ArcGIS software 10.5. Those factors were also collected in the present study.

### 2.4. Outcome: Walking Behavior 

The study focused on general walking behavior. International Physical Activity Questionnaire (IPAQ) was used to assess different domains of walking [51]. The questionnaire was conducted through interviews, ensuring no data were missing.

The following two variables were used as outcomes of physical activity: (a) transportation walking, (b) recreational walking in minutes per week. Transportation walking refers to walking from place to place for transport purpose, such as going to work places, going shopping, which excludes any walking done solely for recreation, sports, and exercise purpose. The recreational walking refers to walking solely for recreation, sports, and exercise purpose.

### 2.5. Analysis

In this study, multilevel regression models were used to explore the associations of neighborhood type and distance to MTR with walking time, after controlling for other walking-influencing built environments (including population density, land-use mix, and intersection density) and individual-level covariates (including age, sex, and household income). Multilevel modeling can account for the clustering in the walking behaviors of participants in public housing estates. Individual participants (Level 1) were modelled to be clustered within public housing estates (Level 2). Two separated multilevel models were used to predict the transportation walking time and recreational walking time respectively.

All analyses were performed in statistical software R using the lme4 package for fitting and analyzing mixed-effects models. Point estimates (standardized *β*), their 95% confidence intervals, and *p*-values were reported for the two models. Count and percentage were reported for descriptive statistics.

### 2.6. Results

**Figure 4:**
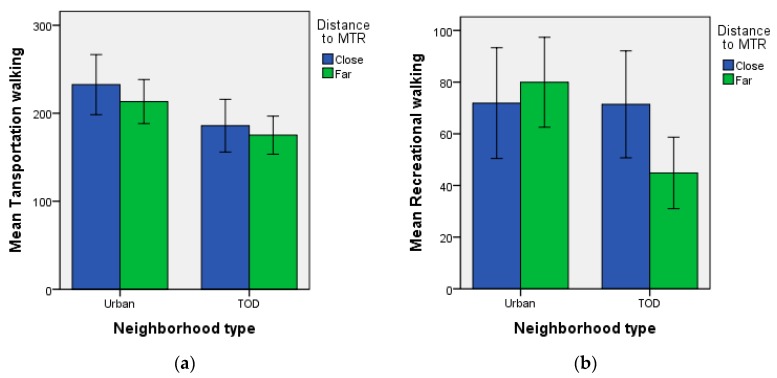
The mean transportation walking (**a**) and recreational walking time (**b**) (in minutes/7 days) by neighborhood types (urban vs. TOD) and distance to MTR stations (close vs. far). The error bars represent standard errors. Public housing residents within 400 m walking distance to a MTR station were classified in the close group; those within 800–1200 m were classified in the far group.

The descriptive statistics of individual variables are presented in Table 1. There were more younger adults (18–34 years) than the other two age group (35–49 years, and 50–64 years). There were slightly more female participants than male. The medium household income constituted the largest income group with 37.4% of participants. The mean transportation and recreational walking time by neighborhood types and distance to MTR stations were shown in Figure 4.

The results of multilevel regression model for predicting walking time are shown in Table 2. For transportation walking time, the main effect of neighborhood types was significant, while the main effect of distance to MTR or interaction effect between neighborhood type and distance to MTR was not significant. The results indicated that public housing residents living in urban neighborhoods had significantly more transportation walking compared with those in TOD neighborhoods (Figure 4).

For recreational walking, the main effect of neighborhood types and distance to MTR was not significant, while the interaction effect between neighborhood type and distance to MTR was significant (Table 2). The post-hoc simple effects analysis revealed that the TOD residents living far from MTR stations had significantly less recreational walking compared with TOD residents living close to MTR (Figure 4), (*β* (95% CI) = −0.57 (−1.07, −0.07), *p* = 0.03). The urban residents living far from MTR stations has similar level of creational walking compare with urban residents living close to MTR (*β* (95% CI) = 0.17 (−2.92, 3.25), *p* = 0.62).

## 3. Discussion

This study compared 616 residents’ walking behaviors of a total of 20 traditional urban and TOD neighborhoods in Hong Kong. The study could reduce residence self-selection bias by focusing on residents who had been largely assigned to public housing estates based on flat availability rather than their individual preferences. After controlling for potential confounding variables—including land use mix, population density, street intersection density, and individual variables, the study detected some differences in transportation and recreational walking by neighborhood types (traditional urban vs. new town) and distance to MTR stations (close vs. far).

Similar jobs-housing imbalance has been reported in other Asian cities adopting TOD concept in new town design, e.g., Beijing, Shanghai, and Seoul [35,38,39,40,41]. The rail-transit expansions have often accompanied by housing development, but not the jobs and businesses per se. Those TOD new towns along rail lines have largely become bedroom communities.

We found that new town residents had less transportation walking compared to urban residents. The possible reason is TOD had relatively fewer pedestrian destinations, such as retail outlets and services than established urban neighborhoods. Furthermore, there is a strong spatial clustering of those destinations around the MTR stations.

We also found that new town residents living close to MTR had similar transportation walking compared to with those living far from MTR stations. We interpret that new town residents who are far from MTR stations may make longer transportation walking, such as shopping or visiting a clinic. This is primarily because residents living far from MTR walk longer from home to reach those destinations (retails and services) comparing those living close to MTR as those destinations are often concentrated around MTR stations in new towns. The finding can be further interpreted in the light of pre-existing local social context; Hong Kong has well-developed public transportation systems, and low private car ownership in contrast to most Western cities. Thus, residents living far from MTR still heavily rely on walking or public transit for transportation trips in Hong Kong.

Recreational walking showed the biggest spatial disparity. The new town residents who were far from transit station had about significantly less recreational walking compared with TOD residents close to transit stations.

The variation of recreational walking can be accounted for by the spatial disparity of pedestrian sidewalks and recreational facilities—including parks, sports courts, and physical activity facilities. We purposely selected the housing estates on the outskirts of new towns into the far-away-from-MTR-station group. Those public housing estates, has less well-connected and well-maintained pedestrian sidewalks in the neighborhoods since the sidewalks often connected to the MTR station, but not necessarily to the surrounding open rural areas confining new towns. Furthermore, new town neighborhoods generally had less recreational facilities (M = 21.2, SD = 12.2) comparing with urban neighborhoods (M = 31.8, SD = 18.7). Therefore, the lack of street connection and of recreational facilities, in conjunction with other factors such as immense size of street block, enclosed housing estates, hierarchical road system, the pedestrian system organized according to the motorized road system—which is circuitous and designed for efficient traffic flow—may explain the observed drop of recreational walking for the peripheral new town residents.

In sum, this study demonstrated that TOD-featured new town neighborhoods and established urban neighborhoods had different impacts on resident’s walking behavior, even though they share many pedestrian-friendly design characteristics. Compared with urban residents, the new town residents who live close to transit stations arguably had similar level of walking. The new town residents who live far from transit stations, however, benefit less from pedestrian-friendly design characteristics. They had lower level of recreational walking, compared with new town residents who living close to transit stations.

The observed differences in travel behaviors challenges the common assumption that TOD neighborhood and urban neighborhood have similar impact on walking or physical activity. The planning principles of Hong Kong’s new towns is to create high density, mixed use, and pedestrian-friendly urban forms around a transit station, and to confine those urban developments with open rural spaces. This design principle creates urban density profiles that are characterized by substantial spatial disparities in job opportunity, pedestrian destinations, and pedestrian infrastructures within new towns, with an evident disadvantage for residents located on the periphery of new towns. Established urban areas of Hong Kong were developed before the construction of transit lines. The urban area in Kowloon was much larger than any single new town, and many neighborhoods and districts are physically connected. Furthermore, the pedestrian destinations and gridiron street network are evenly distributed through the whole urban area. The spatial homogeneity of pedestrian destinations and infrastructures in urban areas may explain the increased walking for urban residents both close to and far from transit stations. Other built environment differences also explain the difference in walking levels, such as enclosure of housing estates, irregular streets, which may lead to longer walks in new town neighborhoods, compared with small street block and orthogonal layout in traditional urban neighborhoods. Topography may also be an issue, since the old, urban neighborhoods were constructed on flat land and landfill while some new town housing estates are built at higher elevations. More studies are needed to elucidate this issue.

The findings in the present study also have direct policy and design implications for local governments in China. Design policies from Western countries, as currently adopted in Hong Kong, often suggests the TOD should be confined within walking distance (such as 800–1200 m) from transit stations. The observed spatial disparity of walking behaviors in this study suggests that this design strategy may not be as effective as established urban areas.

To enhance walkability potential and promote active living for TOD residents in high-density cities, regional-level policies should promote a more nuanced approach to holistic TOD new town development with adjacent new towns closely clustered together to function as an integrated whole rather than them being *isolated islands of mono-functional bedroom communities* interspersed by surrounding open rural areas. Stress must be laid on the continuity of pedestrian infrastructures and destinations to avoid creation of disjointed clustering of opportunities in relatively small areas (such as around MTR stations) that may over a period promote sedentary behaviors. Last but not the least, there is a necessity for strategic and sustained flows of capital and infrastructural investments to ensure a continuous pattern of development and multi-functional community design as opposed to developments solely focusing on and clustered around MTR stations. Such a pattern will not only create a higher employment density and job mix but also ensure their spatial uniformity, thereby avoiding new towns being proxy for mono-functional bedroom communities with significantly longer commute [35,38,39,40,41,52,53].

## 4. Strength and Limits

This study has several strengths. This is the first study, to our knowledge, to compare TOD-featured new towns and established urban areas in terms of walking behaviors in different domains in Hong Kong, a city often used as an exemplar for implementing TOD principles in many Chinese cities. Another strength is that the models adjusted for objectively assessed built environmental features with GIS data, which ensures the accuracy of our results. To address the issue of residence self-selection bias, we focused on residents living in public housing estates. The research design is essentially a natural experiment. On the other hand, by focusing on public housing residents, the study sampling was exclusive to low-and medium-income people in Hong Kong, and the findings may not apply to the high-income residents. The TOD-featured new towns are also larger and denser than their counterparts in the US, though it is common in other high-density cities. Therefore, the findings in this study may not apply to other low-density cities. The current study only captured self-reported walking time, which is subject to recall bias. Further studies may capture both the frequency and duration of each walking trip with comprehensive walking diary or advanced technology, such as portable global position system (GPS) and accelerometer devices [54].

Further studies are also warranted to disentangle which physical environmental factors considered in this study are associated with which aspects of walking behavior, and to what degree.

## 5. Conclusions

Promoting physical activity is an urgent necessity for Chinese urban residents, given the increasing sedentary behavior and rising risks of obesity and chronic diseases. The concept of transit-oriented development attracts lots of attention for the claimed physical activity benefit compared with old established urban areas. In this study, we compared the residents’ walking behaviors in both new towns and traditional urban neighborhoods of Hong Kong. The results showed that new town residents who reside far from transit stations walked less compared with urban residents. The spatial disparity of pedestrian destinations, jobs and infrastructures may account for the observed difference in walking behavior. The findings challenge the commonly accepted concept that TOD neighborhoods and old traditional urban neighborhoods had similar impacts on residents’ walking behavior, though they share many pedestrian-friendly design characteristics. Further research must focus on the need to further streamline planning and design of new TOD communities taking into account local-level considerations prevalent in Asia with a view to promote active lifestyle and behavior.

## Figures and Tables

**Table 1:** Characteristics of 616 participants in this study (Hong Kong SAR, China in 2016).

Variables	Number of Participants	Percentage (%)
**Age (years)**		
18–34	320	51.9
35–49	165	26.8
50–64	131	21.3
**Gender**		
Male	299	48.5
Female	317	51.5
**Household income (HKD)**		
Low (<12 K)	175	28.8
Medium (12–15 K)	227	37.4
High (>15 K)	205	33.8

**Table 2:** Multilevel regression models for predicting transportation walking and recreational walking time in different public housing estates by the predictors of neighborhood type (urban vs. TOD) and distance to MTR (close vs. far) (Hong Kong SAR, China in 2016, *N* = 616).

Model Predictors	Transportation Walking		Recreational Walking	
	*β* (95% CI)	*p*-value	*β* (95% CI)	*p*-value
**Neighborhood Type**				
Urban-Reference				
TOD	−0.6 (−1.18, −0.03)	0.04	0.43 (−0.22, 1.08)	0.17
**Distance to MTR**				
Close-Reference				
Far	−0.26 (−0.88, 0.35)	0.37	0.52 (−0.18, 1.21)	0.12
Neighborhood Type * Distance to MTR	0.08 (−0.70, 0.86)	0.83	−0.23 (−0.43, −0.03)	0.02

* CI: Confidence Interval. Public housing residents within 400 m walking distance to a MTR station were classified in the close group; those within 800–1200 m were classified in the far group.
